# Rv3400 is a phosphoglucomutase required for trehalose metabolism in *Mycobacterium tuberculosis*

**DOI:** 10.1128/jb.00343-26

**Published:** 2026-09-03

**Authors:** Yi Liu, Nadine Ruecker, Valwynne Faulkner, Kavitha Rachineni, Nada Al-Saffar, Elisabeth Oskoui, Larissa Zarate Garcia, Jessica Emma Rowley, Pablo Villacampa Teixeira, Tiago R. D. Costa, Eachan Johnson, Brian D. Robertson, Sabine Ehrt, Gerald Larrouy-Maumus

**Affiliations:** 1 Department of Life Sciences, Faculty of Natural Sciences, Centre for Bacterial Resistance Biology, Imperial College London 4615, London, United Kingdom; 2 Department of Microbiology and Immunology, Weill Cornell Medicine 12295, New York, New York, USA; 3 Systems Chemical Biology of Infection and Resistance Laboratory, Francis Crick Institute 376570, London, United Kingdom; 4 Section of Bioanalytical Chemistry, Division of Systems Medicine, Department of Metabolism, Digestion and Reproduction, National Phenome Centre, Imperial College London 4615, London, United Kingdom; 5 Flow Cytometry Facility, Department of Life Sciences, Faculty of Natural Sciences, Imperial College London 4615, London, United Kingdom; 6 Department of Infectious Disease, Imperial College London 4615, London, United Kingdom; University of Notre Dame, Notre Dame, Indiana, USA

**Keywords:** metabolomics, trehalose, *Mycobacterium tuberculosis*, glycolysis, enzyme

## Abstract

*Mycobacterium tuberculosis* (Mtb) causes over 1 million deaths from tuberculosis (TB) every year and remains a major burden on human health. Reducing the deadly impact of TB requires a better understanding of the strategies used by *M. tuberculosis* to adapt its metabolism, survive, and persist in the human host. Previous enzymological studies reported that the Mtb *rv3400* gene encodes a β-phosphoglucomutase; however, its role in *M. tuberculosis* metabolism had not been investigated. Here, we show that deletion of *rv3400* causes a 30-fold increase in β-D-glucose-1-phosphate, confirming its primary function as a β-phosphoglucomutase. Deletion of *rv3400* also causes a growth defect when trehalose is the sole carbon source. Targeted metabolomics revealed that metabolites associated with redox homeostasis, including ergothioneine, mycothiol, and mycothione, are decreased in the Mtb Δ*rv3400* strain compared with the wild type, indicating altered tolerance to redox stress. Consistent with this, the Δ*rv3400* strain showed increased susceptibility to oxidative stress induced by H_2_O_2_ and cumene hydroperoxide, conditions that Mtb encounters during infection. This work advances our understanding of trehalose metabolism in Mtb and suggests that Rv3400 may represent a target for the development of new antimicrobial therapies.

**IMPORTANCE:**

Trehalose metabolism plays a cornerstone role in *Mycobacterium tuberculosis* physiology and virulence. A better understanding of the metabolism of this essential disaccharide is therefore required to develop new strategies to eradicate tuberculosis. Here, we characterize *M. tuberculosis* lacking the β-phosphoglucomutase encoded by *rv3400*. We show that deletion of *rv3400* leads to accumulation of β-D-glucose-1-phosphate, impaired growth when trehalose is used as the sole carbon source, and increased susceptibility to oxidative stress. Taken together, these data provide evidence that Rv3400 is required for optimal catabolism of trehalose.

## INTRODUCTION

*Mycobacterium tuberculosis* (Mtb) causes over 1 million deaths annually and remains a major global health threat (1). Although antibiotics have been used for decades, drug resistance and persistent infections severely limit current treatments against this global killer. A key challenge in combating Mtb is understanding how it adapts to host environments, such as macrophages, by reprogramming its metabolism and gene expression. Addressing this knowledge gap is important for identifying new druggable targets and achieving the WHO’s tuberculosis eradication goals through next-generation therapeutics.

**Fig 1:**
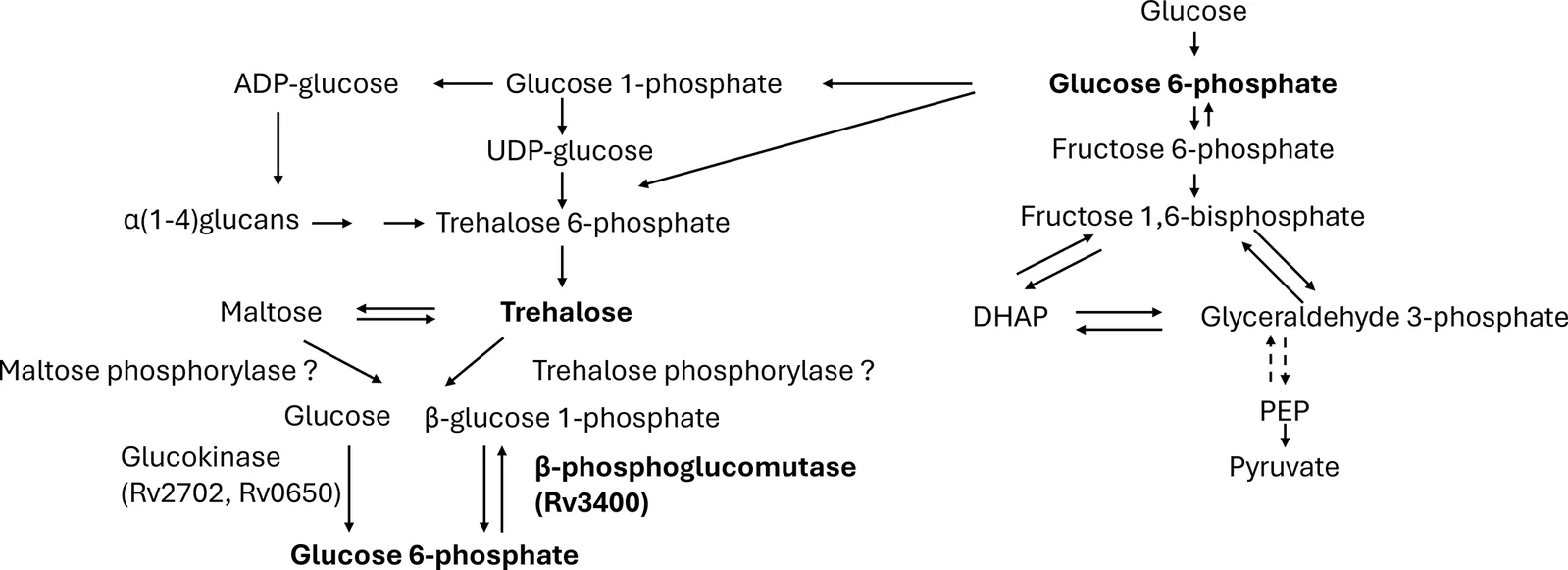
Scheme presenting the trehalose metabolism pathway.

Recent *in vitro* studies have characterized Rv3400 as a β-phosphoglucomutase (β-PGM), an enzyme that catalyzes the isomerization of β-D-glucose-1-phosphate (G1P) to β-D-glucose-6-phosphate (G6P) via a β-D-glucose-1,6-bisphosphate (β-G1,6-bisP) intermediate (2) (Fig. 1). The enzyme is closely linked to central carbon metabolism, as G6P serves as a key metabolic node to produce ATP and NADPH via glycolysis and the pentose phosphate pathway, respectively (3). Although Rv3400 is dispensable for *in vitro* growth of Mtb H37Rv (4–6), it is required for bacterial growth in the spleen of C57BL/6J mice (5, 6). Furthermore, using genetic interaction mapping, Joshi et al. reported the interaction between Rv3400 and the virulence-associated mycobacterial cell entry 4 (*mce4*) operon, suggesting a potential role in Mtb virulence (7).

Recent studies also suggest that Rv3400 could be part of the trehalose degradation pathway (2) (Fig. 1). Trehalose is a non-reducing disaccharide (1-O-α-D-glucopyranosyl-α-D-glucopyranoside) found in insects, plants, and microbial cells. In mycobacteria, trehalose is constitutively present in cells grown in standard laboratory media and is the only free sugar readily detectable in the cytoplasm, comprising approximately 1.5%–3% of the total cellular dry weight (8). Trehalose has well-established protective functions during cryopreservation and desiccation and is known to enhance microbial survival under environmental stress *in vivo*. In mycobacteria, trehalose is not only a reserved energy source but also an active player in metabolic pathways, as suggested by the dynamic turnover of the trehalose pool found in the rapid-growing mycobacteria *Mycobacterium smegmatis* (9). It is also incorporated into many mycobacterial glycolipids, including cord factor (trehalose 6,6′-dimycolate), sulfolipids (acylated trehalose-2′-sulfate derivatives), and trehalose-containing lipooligosaccharides (10–12). These multiple roles further underscore the importance of trehalose metabolism in Mtb survival and pathogenesis.

Here, we investigated the impact of *rv3400* deletion on Mtb metabolism. We found that the Mtb Δ*rv3400* strain has a growth defect when trehalose is used as the carbon source and accumulates β-D-glucose-1-phosphate, consistent with the loss of β-PGM activity. We further show that *rv3400* deletion alters carbon metabolism and redox metabolites, leading to increased susceptibility to oxidative stress.

## RESULTS AND DISCUSSION

### Deletion of *rv3400* leads to an increase in β-D-glucose-1-phosphate level

**Fig 2:**
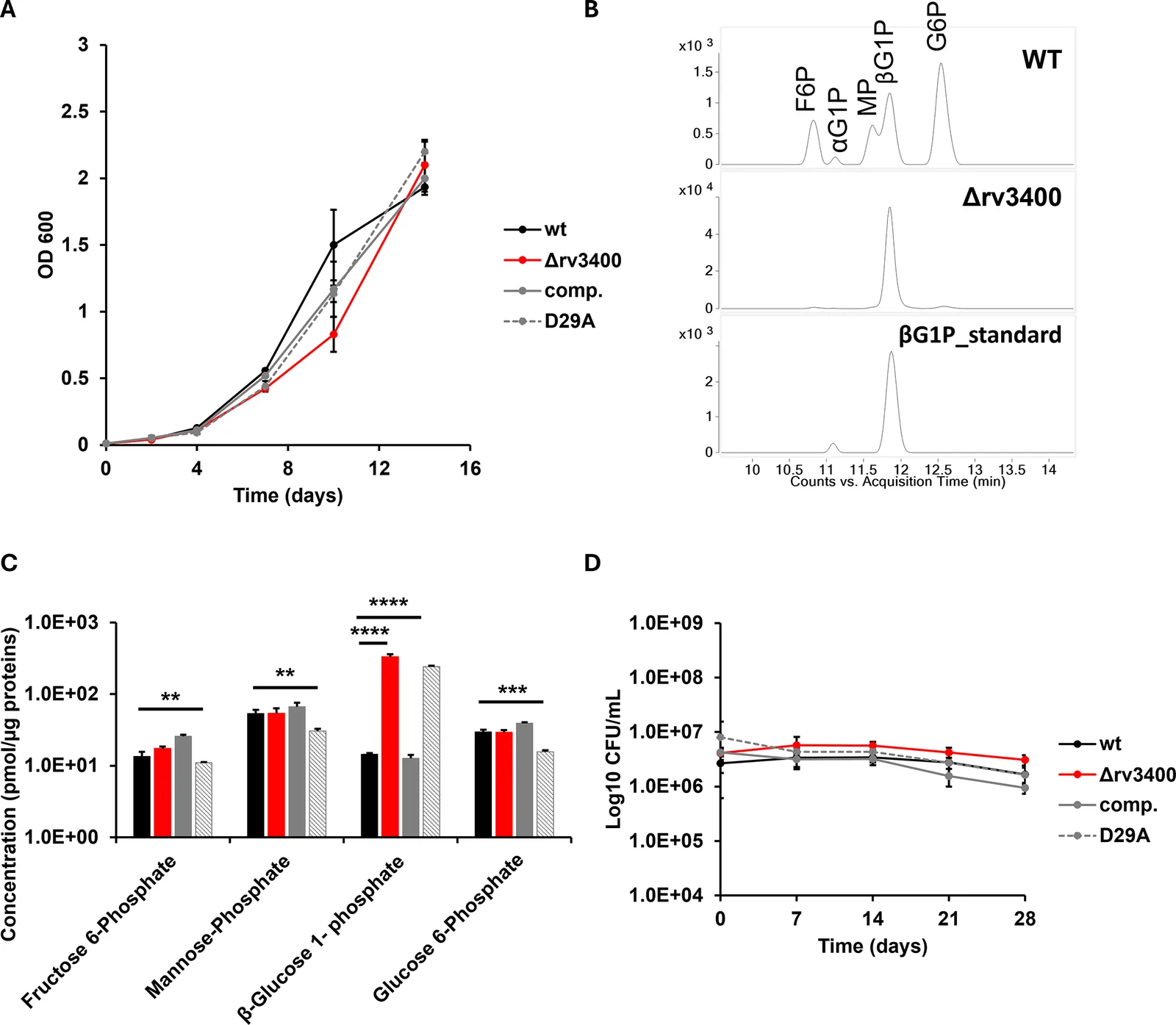
Rv3400 is a phosphoglucomutase in *Mycobacterium tuberculosis*. (**A**) Growth curves of wild-type (black) and Δ*rv3400* (red) strains with glucose as the sole carbon source. (**B**) Extraction ion chromatograms for ions at *m*/*z* 259.024 [M–H]^−^ for the wild-type (top), Δ*rv3400* strains (middle), and β-D-glucose-1-phosphate standard (bottom). (**C**) Bar charts displaying the concentration in pmol/µg proteins for the five relevant phosphorylated sugars (F6P: fructose-6-phosphate; G1P: glucose-1-phosphate; βG1P: β-D-glucose-1-phosphate; MP: mannose-6-phosphate/mannose-1-phosphate; and G6P: glucose-6-phosphate). Wild type: black bars, Δ*rv3400*: red bars, complemented: gray bars, and D29A: shaded gray bars. (**D**) Survival assay under PBS starvation as measured by the number of viable cells (CFU/mL). Asterisks indicate levels of significance compared to wild-type strain bacteria. The significance level of differences was determined by unpaired Student’s *t*-tests, with *, *P* < 0.05; **, *P* < 0.01; ***, *P* < 0.001; ****, *P* < 0.0001.

Recent work has identified Rv3400 as a β-PGM *in vitro* (2). To confirm its enzymatic function, we constructed the *rv3400* deletion mutant (Δ*rv3400*), complemented (Δ*rv3400*::*rv3400*), and catalytic site mutant (Δ*rv3400*::*rv3400*_D29A_) strains and measured their growth in carbon-defined liquid medium. As shown in Fig. 2A, compared to the wild-type strain, deletion of *rv3400* did not impair growth when glucose was used as the sole carbon source. To further validate the role of Rv3400 as a β-PGM, we used liquid chromatography-mass spectrometry (LC-MS) analysis to extract the ion chromatograms for the ion at *m*/*z* 259.024 [M–H]^−^, assigned to monosaccharide phosphates: fructose 6-phosphate (retention time [RT], 11.1 min), glucose-1-phosphate (RT, 11.3 min), mannose-1-phosphate/mannose-6-phosphate (RT, 11.6 min), β-glucose-1-phosphate (RT, 11.8 min), and glucose-6-phosphate (RT, 12.4 min). In the Δ*rv3400* strain, the peak corresponding to β-D-glucose-1-phosphate (RT, 11.8 min) increased (Fig. 2B). The assignment of this peak to β-D-glucose-1-phosphate was confirmed by nuclear magnetic resonance (NMR) (Supplemental text and Fig. S1 to S6). In addition, quantification of the four monosaccharide phosphates revealed that *rv3400* deletion specifically elevated the concentration of β-glucose-1-phosphate (Fig. 2C), confirming that Rv3400 is a β-PGM involved in the conversion of β-D-glucose-1-phosphate into β-D-glucose-6-phosphate. Interestingly, the levels of the four other monosaccharide phosphates—fructose 6-phosphate, mannose 1-phosphate/6-phosphate, and glucose 6-phosphate—were not altered in the Δ*rv3400* compared to the wild type (Fig. 2C).

To gain further insights into how the Δ*rv3400* maintains monosaccharide phosphate levels similar to those of the wild type, we performed qRT-PCR on the two glucokinase genes *rv2702* and *rv0650*. As shown in Table S1, no significant differences were observed between the wild type and Δ*rv3400* as well as Δ*rv3400*::*rv3400*_D29A_. To investigate this observation further, we performed stable isotope labeling using [U-^13^C_6_]-glucose. This experiment tested whether carbon metabolism differed in Δ*rv3400* compared to the wild type. As shown in Table S2, no changes in the ^13^C incorporation were observed for either fructose 6-phosphate or glucose 6-phosphate. However, ^13^C incorporation into β-glucose-1-phosphate was markedly decreased in Δ*rv3400* and Δ*rv3400*::*rv3400*_D29A_ compared to the wild type, with approximately ~25% compared to ~60%, respectively.

Together, these experiments indicate that deletion of *rv3400* impairs the conversion of β-D-glucose-1-phosphate into glucose 6-phosphate (Fig. 1) without affecting the levels of the other monosaccharide phosphates, as the turnover of both fructose 6-phosphate and glucose 6-phosphate was not altered by *rv3400* deletion.

Because glucose phosphorylation is not essential for long-term PBS starvation (13), we next investigated the effect of prolonged PBS starvation on the survival of the Mtb Δ*rv3400* strain. As shown in Fig. 2D, Rv3400 was not required for bacterial viability during starvation, consistent with previous literature (13). Measurement of reactive oxygen species (ROS) after 28 days of PBS starvation using CellROX Deep Red showed no difference in ROS production between the wild-type and Δ*rv3400* strain (Fig. S6), confirming that Rv3400 is dispensable under long-term PBS starvation.

### Deletion of *rv3400* impairs growth when trehalose is used as the sole carbon source

**Fig 3:**
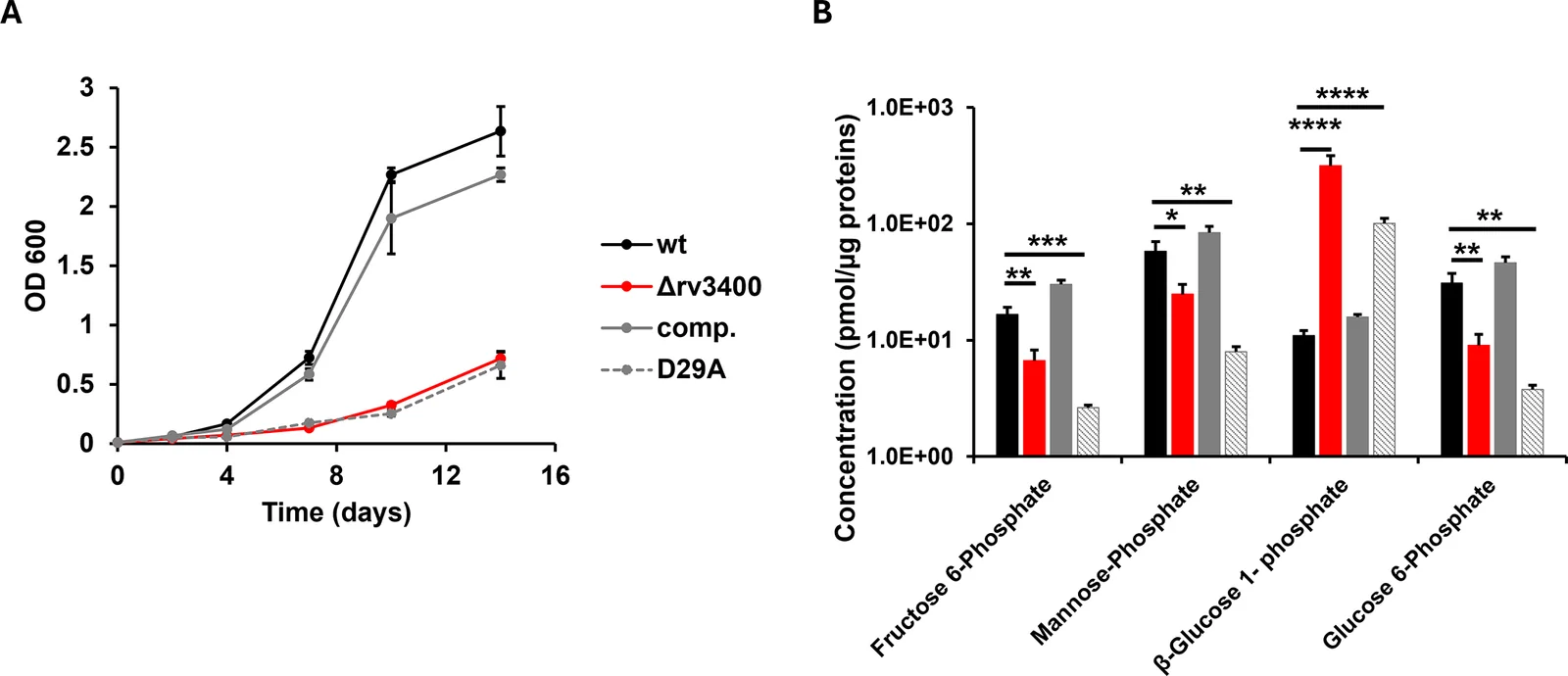
Rv3400 is required for trehalose recycling. (**A**) Growth curves of wild-type (black), Δ*rv3400* (red), complemented (gray), and D29A (dashed gray) strains with trehalose as the sole carbon source. (**B**) Bar charts displaying the concentration in pmol/µg proteins for the four relevant phosphorylated sugars (F6P: fructose-6-phosphate; βG1P: β-D-glucose-1-phosphate; MP: mannose-6-phosphate/mannose-1-phosphate; and G6P: glucose-6-phosphate). Wild type: black bars, Δ*rv3400*: red bars, complemented: gray bars, and D29A: shaded gray bars.The significance levels of difference were determined by unpaired Student’s t-tests, with *, *P* < 0.05; **, *P* < 0.01; ***, *P* < 0.001; ****, *P* < 0.0001.

The trehalose recycling pathway mediated by the LpqY-SugA-SugB-SugC ATP-binding cassette transporter is critical during acute Mtb infection in the mouse model, when exponential replication occurs and trehalose can serve as a carbon source; disruption of this pathway may cause starvation in nutrient-limited *in vivo* microenvironments (14, 15). Given the proposed role of Rv3400 in trehalose recycling, we assessed the growth of Δ*rv3400* and Δ*rv3400*::*rv3400*_D29A_ strains with trehalose as the sole carbon source. As shown in Fig. 3A, both strains exhibited growth defects under this condition.

To further support this observation, we performed growth curves using 0.05%, 0.02%, and 0.4% glucose or trehalose. As expected, glucose was used as the sole carbon source, and increasing glucose concentration increased the growth of the wild-type, Δ*rv3400,* and complemented strains. However, when trehalose was used as the sole carbon source, growth increased with higher trehalose concentrations in the wild-type and complemented strains, whereas no increase in growth was observed for Δ*rv3400* when more carbon was available in the culture medium (Fig. S7). To rule out contaminants that could interfere with bacterial growth, such as fatty acids originating from bovine serum albumin (BSA) fraction V, we repeated the experiment using fatty-acid-free BSA fraction V. A similar result was observed: the Δ*rv3400* strain did not display increased growth with increasing trehalose concentration (Fig. S8).

Targeted metabolomics further revealed that the Δ*rv3400* mutant accumulated a 30-fold higher β-glucose-1-phosphate pool, while showing twofold, twofold, and threefold reductions in fructose 6-phosphate, mannose 1-phosphate/6-phosphate, and glucose 6-phosphate pools, respectively (Fig. 3B). A similar trend is observed in the metabolic profile of the D29A strain (Fig. 3B). Taken together, these data demonstrate that Rv3400 is required for optimal use of trehalose in Mtb.

Because bacterial growth represents only one aspect of bacterial metabolism, we next performed LC-MS metabolomics analysis of the intracellular metabolome of each strain. We selected a set of 36 metabolites belonging to central carbon metabolism, the tricarboxylic acid cycle, amino acid metabolism, nucleotide metabolism, redox metabolism, and peptidoglycan metabolism (Table S3).

**Fig 4:**
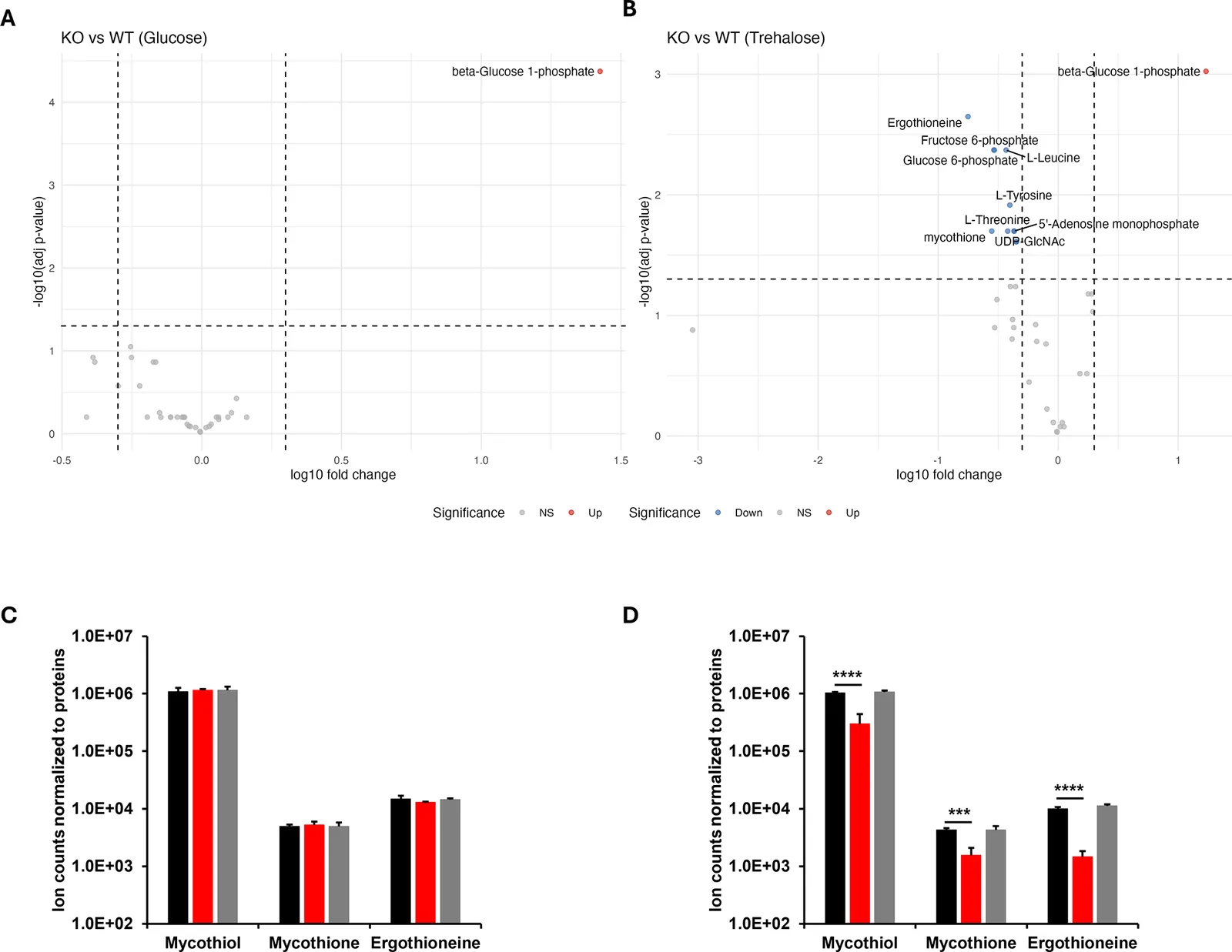
Deletion of *rv3400* impairs the biosynthesis of redox metabolites. (**A**) Volcano plots of targeted metabolites for strains grown in glucose as the sole carbon source. (**B**) Volcano plots of targeted metabolites for strains grown in trehalose as the sole carbon source. (**C**) MSH, MSSM, and ergothioneine levels for the wild-type (black), Δ*rv3400* (red), and complemented (gray) strains when using glucose as the sole carbon source. (**D**) MSH, MSSM, and ergothioneine levels for the wild-type (black), Δ*rv3400* (red), and complemented (gray) strains when using trehalose as the sole carbon source. Asterisks indicate levels of significance compared to wild-type strain bacteria. The significance level of differences was determined by unpaired Student’s *t*-tests, with *, *P* < 0.05; **, *P* < 0.01; ***, *P* < 0.001; ****, *P* < 0.0001. Volcano plots display log10 fold change against −log10(adjusted p-value), with thresholds indicated by dashed lines, and the most significant metabolites were annotated based on adjusted *p*-values.

Consistent with the observation that *rv3400* deletion does not strongly affect the global metabolome when glucose is used as the sole carbon source, the volcano plot identified only one significantly increased metabolite, β-glucose-1-phosphate, in the Δ*rv3400* mutant strain compared with the wild type (Fig. 4A).

However, when trehalose was used as the sole carbon source, 12 of the 36 metabolites showed significant changes in the Δ*rv3400* mutant strain compared to the wild type (Fig. 4B). Notably, the redox metabolites ergothioneine and mycothione showed some of the largest decreases in the Δ*rv3400* mutant. Using targeted LC-MS metabolomics, we measured the intracellular levels of mycothiol (MSH), mycothione (MSSM), a metabolite crucial for maintaining intracellular redox homeostasis in mycobacterial cells (16), and ergothioneine, a sulfur-containing histidine derivative with potent antioxidant and redox-buffering properties (17–20), in the wild-type, Δ*rv3400*, and complemented strains. No changes in MSH, MSSM, or ergothioneine levels were observed when the wild-type, Δ*rv3400,* and complemented strains were grown on glucose as the sole carbon source (Fig. 4C). By contrast, when grown on trehalose as the sole carbon source, the Δ*rv3400* strain showed an approximately threefold decrease in both MSH and MSSM and an approximately 10-fold decrease in ergothioneine compared with the wild type (Fig. 4D). These results indicate that Rv3400-dependent trehalose metabolism is important for maintaining bacterial redox homeostasis.

### Rv3400 is required to maintain redox balance when trehalose is used as the sole carbon source

**Fig 5:**
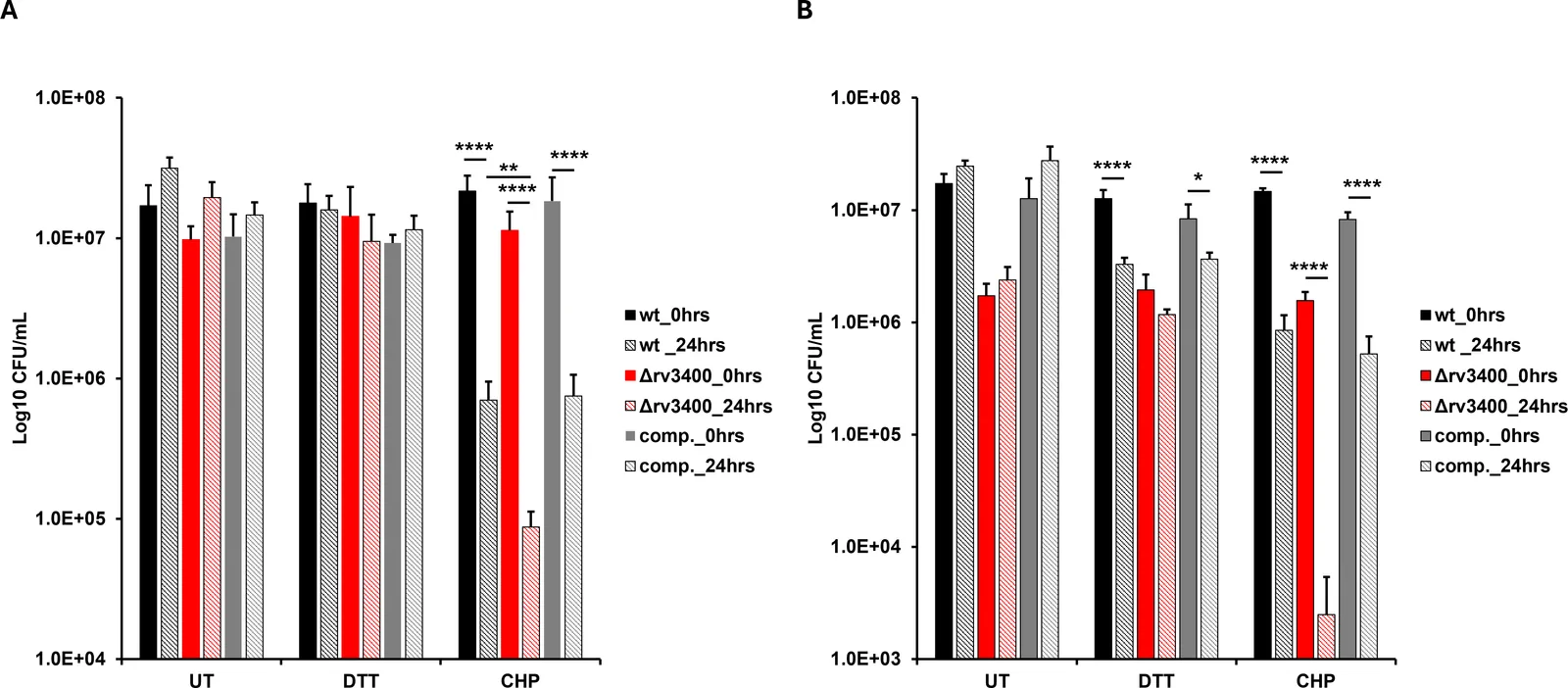
Deletion of *rv3400* leads to an increase in susceptibility to oxidative stress. (**A**) Bar charts displaying the number of viable bacteria expressed as CFU/mL for the wild type: black bars, Δ*rv3400*: red bars, complemented: gray bars at time 0 h for untreated (UT), DTT, or CHP-treated bacteria, and for the wild type: dashed black bars, Δ*rv3400*: dashed red bars, complemented: dashed gray bars at time 24 h for UT, DTT, or CHP-treated bacteria, when using glucose as the sole carbon source. (**B**) Bar charts displaying the number of viable bacteria expressed as CFU/mL for the wild type: black bars, Δ*rv3400*: red bars, complemented: gray bars at time 0 h for UT, DTT, or CHP-treated bacteria, and for the wild type: dashed black bars, Δ*rv3400*: dashed red bars, complemented: dashed gray bars at time 24 h for UT, DTT, or CHP-treated bacteria, when using trehalose as the sole carbon source. Asterisks indicate levels of significance. The significance levels of differences were determined by unpaired Student’s *t*-tests, with *, *P* < 0.05; **, *P* < 0.01; ***, *P* < 0.001; ****, *P* < 0.0001.

Based on the metabolomics data (Fig. 4D), which showed that MSH, MSSM, and ergothioneine levels were decreased in the Δ*rv3400* mutant grown on trehalose as the sole carbon source, we hypothesized that this strain would have compromised redox balance. To test this hypothesis, we first investigated the viability of the wild-type, Δ*rv3400,* and complemented strains after 24 h exposure to reductive stress (dithiothreitol, DTT) or oxidative stress (cumene hydroperoxide, CHP) using either glucose or trehalose as the sole carbon source. When grown in glucose as the sole carbon source, no changes in CFU/mL were observed across strains exposed to DTT (Fig. 5A). Exposure to CHP caused an approximately 1 log_10_ decrease in viable bacteria in the wild-type strain and an approximately 2 log_10_ decrease in the Δ*rv3400* strain, suggesting that *rv3400* deletion renders bacteria more susceptible to oxidative stress. When grown in trehalose as the sole carbon source (Fig. 5B), exposure to DTT led to an approximately 0.3 log_10_ reduction in viable bacteria in the wild-type strain, whereas no change in CFU was observed in the Δ*rv3400* strain. Following CHP treatment, an approximately 1 log_10_ decrease in viable bacteria was observed in the wild-type strain, and an approximately 3 log_10_ decrease was observed in the Δ*rv3400* strain. A similar phenotype was observed using H_2_O_2_ as a more physiological *in vitro* oxidative stressor. Although no changes in CFU were observed when glucose was used as the sole carbon source, the Δ*rv3400* strain showed an approximately 1 log_10_ decrease in CFU compared with the wild type when trehalose was used as the sole carbon source (Fig. S9), confirming that the Δ*rv3400* strain is more susceptible to oxidative stress than the wild type.

Taken together, our data demonstrate that Rv3400 is a β-PGM required for optimal trehalose utilization when trehalose is present as the sole carbon source. This study provides in-cell validation of Rv3400 function in Mtb trehalose metabolism and highlights its contribution to bacterial redox homeostasis. The trehalose synthesis and recycling pathway has been studied for its involvement not only in Mtb regrowth but also in the formation of persisters and the development of multidrug resistance (21, 22). Therefore, our study on Rv3400 contributes substantially to understanding this pathway as an important part of Mtb metabolism.

## MATERIALS AND METHODS

### Bacterial strains and growth conditions

The Mtb Δ*rv3400* mutant was constructed in strain H37Rv by allelic exchange using a recombineering approach as previously described (23).

A pMV306 plasmid with the *rv3400* native promoter was used as the backbone for construction of the *rv3400* complementation plasmid. To generate a complementation of *rv3400* under its native promoter, the integrating expression vector pMV306 KanR (24) was digested with EcoRV-HF (NEB) and subsequently column purified using the QIAquick purification kit (Qiagen). DNA fragments comprising the full length of *rv3400* and *rv3400* D29A along with 150 bp upstream of the annotated start codon were synthesized (GenScript). The synthesized fragments included 20 bp overhangs at both the 5′ and 3′ ends homologous to the digested vector. NEBuilder HiFi DNA assembly (NEB) was used to ligate the linearized vector and synthesized inserts. Correct assembly of the recombinant plasmids was confirmed by Nanopore sequencing.

Precultures of all strains were grown in 30 mL square bottles containing 10 mL 7H9 medium (DIFCO) supplemented with 0.2% wt/vol glucose, 0.5% wt/vol standard BSA, 0.08% wt/vol NaCl, 0.05% wt/vol tyloxapol, and appropriate antibiotics. The bottles were incubated in a shaking incubator at 37°C and 120 rpm. 7H10 agar plates (Sigma) were supplemented with 0.2% wt/vol glycerol, 0.2% wt/vol dextrose, 0.5% wt/vol BSA, and 0.08% wt/vol NaCl for growth on solid medium in a 37°C static incubator. For carbon source concentration dependence, fatty-acid-free BSA fraction V was used, while carbon source concentrations were either 0, 0.05%, 0.2%, or 0.4% final concentration wt/vol.

### Growth curves

Growth curve assays were performed in a 30 mL square bottles (Nalgene) containing 10 mL pre-warmed 7H9 (DIFCO) medium. Precultures of bacteria were grown and used to inoculate these flasks at OD_600nm_ = 0.01. Inoculated flasks were incubated in a 37°C static incubator, and OD_600nm_ measurements were taken at least five times to produce growth curves until the stationary phase.

### Bacteria viability under starvation

To quantify the number of viable cells under PBS starvation, mid-log phase cultures were spun down, washed twice with PBS, and resuspended at an OD_600nm_ = 0.1 in PBS 0.5% tyloxapol. Cultures were sampled on days 0, 7, 14, 21, and 28 and plated on 7H10 agar plates. Colonies were counted after 3–4 weeks of incubation at 37°C.

### Bacteria viability under redox stresses

To quantify the number of viable cells under redox stresses, mid-log phase cultures were exposed to 10 mM DTT, 400 µM CHP, for 24 h or 10 mM H_2_O_2_ for 1 h. Cultures were sampled at day 0 and 24 h after exposure for DTT and CHP, and after 1 h for H_2_O,_2_ and plated on 7H10 agar plates. Colonies were counted after 3–4 weeks of incubation at 37°C.

### Metabolite extraction

Mtb strains were grown in 10 mL flasks with 7H9 medium to reach OD_600nm_ of approximately 0.8. After incubation, bacteria were collected by centrifugation at 3,000 × *g*, 4°C for 10 min. Pellets were washed once with cold sterile PBS and metabolically quenched in extraction solution (40% acetonitrile, 40% methanol, and 20% ddH_2_O). Samples were transferred to microtubes containing 0.1 mm acid-washed zirconia beads and lysed using a FastPrep-24 homogenizer twice at 6.0 m/s for 30 s, with a 5 min interval. Supernatants were transferred to 0.22 μm spin X column filters (Costar) and filtered twice by centrifuging at 15,000 × *g* for 30 min. The bacterial biomass of the individual samples was determined by measuring the residual protein content of the metabolite extracts using the BCA assay kit (Thermo) (25, 26). After filtration, the flowthrough was mixed with an equal volume of acetonitrile and centrifuged at 15,000 × *g* for 10 min. A total of 100 µL of the mixture was loaded into polypropylene snap vials (Agilent Technologies) for LC-MS analysis.

### Metabolite extraction with stable isotope (^13^C) labeling

[U-^13^C_6_] glucose was purchased from Cambridge Isotope Laboratories, UK. ^13^C-labeled 7H9 medium was prepared by substituting dextrose in the supplement with [U-^13^C_6_] glucose at the same concentration. Mtb strains were grown in 10 mL flasks with 7H9 medium to reach OD_600nm_ of approximately 0.6–0.8. On the day of labeling, bacteria were collected by centrifugation and resuspended in 5 mL ^13^C-labeled 7H9. Labeled cultures were incubated in a shaking incubator at 37°C for 4 h to allow label incorporation. After incubation, bacteria were collected by centrifugation at 3,000 × *g* for 10 min at 4°C. Pellets were washed once with cold sterile PBS and metabolically quenched in extraction solution (40% acetonitrile, 40% methanol, and 20% ddH_2_O). Samples were transferred to microtubes containing 0.1 mm acid-washed zirconia beads and lysed using a FastPrep-24 homogenizer twice at 6.0 m/s for 30 s, with a 5 min interval. Supernatants were transferred to 0.22 μm spin X column filters (Costar) and filtered twice by centrifuging at 15,000 × *g* for 30 min. After filtration, the flowthrough was mixed with an equal volume of acetonitrile and centrifuged at 15,000 × *g* for 10 min. A total of 100 µL of the mixture was loaded into polypropylene snap vials (Agilent Technologies) for LC-MS analysis.

### LC-MS

The data were acquired with an Agilent 1290 Infinity II UHPLC coupled to a 6545 LC/Q-TOF system. Chromatographic separation was performed with an Agilent InfinityLab Poroshell 120 HILIC-Z (2.1 × 100 mm, 2.7 μm [p/n 675775-924]) column. The HILIC-Z methodology was optimized for polar acidic metabolites. The column compartment was set at 50°C. For easy and consistent mobile-phase preparation, a concentrated 10× solution consisting of 100 mM ammonium acetate (pH 9.0) in water was prepared to produce mobile phases A and B. Mobile phase A consisted of 10 mM ammonium acetate in water (pH 9) with a 5 μM Agilent InfinityLab deactivator additive (p/n 5191-4506), and mobile phase B consisted of 10 mM ammonium acetate (pH 9) in 10:90 (vol:vol) water/acetonitrile with 5 μM Agilent InfinityLab deactivator additive (p/n 5191-4506). The following gradient was applied at a flow rate of 0.25 mL/min: 0 min, 96% B; 2 min, 96% B; 5.5 min, 88% B; 8.5 min, 88% B; 9 min, 86% B; 14 min, 86% B; 17 min, 82% B; 23 min, 65% B; 24 min, 65% B; 24.5 min, 96% B; 26 min, 96% B, and 3 min of re-equilibration at 96% B. Accurate MS was performed using an Agilent Accurate Mass 6545 QTOF apparatus. Dynamic mass axis calibration was achieved by continuous infusion after the chromatography of a reference mass solution using an isocratic pump connected to an electrospray ionization source operated in negative-ion mode. The following parameters were used: gas temperature, 225°C; drying gas, 13 L/min^−1^; sheath gas temperature, 350°C; nebulizer pressure, 35 psi; sheath gas flow, 12 L/min^−1^; capillary voltage, 3,500 V; nozzle voltage, 0 V; fragmentor voltage, 125 V; skimmer 45 V, and octupole 1 RF voltage, 750 V. The data were collected in centroid 4 GHz (extended dynamic range) mode.

### LC-MS metabolomics data analysis

MassHunter Qualitative Analysis software was used to check the extracted ion chromatograms of the *m*/*z* values of interest and obtain their retention times, which were used to construct the personal compound database and library (PCDL) for following analysis. Standards were purchased from Sigma-Aldrich: α-D-fructose-6-phosphate (F1502-1G), α-D-glucose-6-phosphate (G7250), α-D-glucose-1-phosphate (G7000-5G), Calbiochem α-D-mannose-6-phosphate (444100-50MG), and Biosynth Ltd. for β-D-glucose-1-phosphate (MG02984).

Metabolite data were processed in MetaboAnalyst (v6.0) and R (v4.5.2) using a custom analysis pipeline. Technical replicates were collapsed by averaging values within each biological replicate.

Metabolite matrices (metabolites × samples) were constructed for differential analysis data sets. Batch effects attributable to biological repeat were corrected using the ComBat function (sva package v3.58.0), with a design matrix including genetic background and carbon source as covariates to preserve biological signal.

For differential metabolite analysis (volcano plots), linear models were fitted using limma (v3.66.0) on batch-corrected, log_10_-transformed data. Limma fits a linear model to each metabolite and applies empirical Bayes moderation, which improves variance estimation by borrowing information across metabolites and shrinking individual variance estimates toward a common value. Analyses were performed separately for each carbon source, comparing knockout and wild-type strains.

Differential abundance was reported as log_10_ fold change (log_10_FC), consistent with the log_10_ transformation applied to the data. *P* values were adjusted using the Benjamini–Hochberg false discovery rate (FDR). Metabolites were considered significant if the FDR-adjusted *P* value was <0.05 and absolute log_10_FC was >0.3. Volcano plots display log_10_ fold change against −log_10_(adjusted *P* value), with thresholds indicated by dashed lines, and the most significant metabolites were annotated based on adjusted *P* values.

### Stable isotope labeling

Under the experimental conditions described above using [U-^13^C_6_]-glucose (99%), the extent of ^13^C labeling of each metabolite was determined by dividing the summed peak-height ion intensities of all ^13^C-labeled species by the ion intensity of both labeled and unlabeled species using Agilent Profinder version B.8.0.00 service pack 3.

### NMR

Dried extracts of wild-type and knockout bacterial cells were resuspended in 200 µL of a mixture of 90% D_2_O (Sigma-Aldrich) and 10% phosphate buffer (pH 7.4) prepared for NMR analysis as reported in Dona et al. (27). The solution was vortexed for 1 min before centrifugation at 17,000 × *g* for 10 min at 4°C. An aliquot of 180 µL of the supernatant was transferred into a 3 mm NMR tube for NMR analysis.

All NMR spectra were acquired using a Bruker Avance III console combined with a 14.1 T magnet (600 MHz for ¹H NMR). The system was equipped with a 5 mm broadband inverse probe with *z*-axis magnetic field-gradient and a Bruker Sample Jet system, set to 3 mm shuttle mode with a cooling rack maintaining refrigerated tubes at 6°C.

A pre-saturated proton 1D-NOESY pulse sequence was used to obtain 1D ^1^H NMR spectra, with the offset set at 4.70 ppm for water suppression. A total of 65,536 data points were collected with a spectral width of 20.0 ppm and a total of 64 scans (27).

A set of 2D NMR experiments was conducted for structural elucidation of the unknown compound present in the knockout samples, including Jres (J-resolved spectroscopy), ¹H–¹H COSY (correlation spectroscopy), and ¹H–¹H TOCSY (total correlation spectroscopy). All NMR data were processed using TopSpin 3.6 (Bruker BioSpin GmbH, Rheinstetten, Germany). Processing steps included Fourier transformation, phasing, baseline correction, and spectral calibration.

Spiking experiments using an authentic chemical standard were used to further confirm that the unknown compound in the knockout sample was β-D-glucose-1-phosphate. The NMR sample of the knockout cells was spiked with increasing volumes of β-D-glucose-1-phosphate chemical standard (Biosynth Ltd; MG02984) ranging from 0.5 µL to 2 µL, prepared at a concentration of 30 µM in D_2_O, resulting in an increase in the signal intensity of the NMR resonances of interest in the knockout sample.

### ROS measurement using CellROX Deep Red

Cultures after 28 days of starvation in PBS containing 0.5% tyloxapol were harvested by centrifugation (3,000 × *g* for 5 min) and resuspended in 200 µL PBS containing 5 µM CellROX Deep Red (catalog no. C10422; Invitrogen) and incubated at 37°C for 30 min. After incubation, cells were washed to remove residual dye by centrifugation (3,000 × *g* for 5 min). Cells were resuspended in 300 µL PBS containing 4% paraformaldehyde for 1 h at room temperature. Cells were then washed with PBS and resuspended in 200 µL PBS. Fluorescence was measured at a fixed emission (670 nm) after excitation with a red laser (640 nm) using a Cytek Amnis CellStream flow cytometer (Cytek, Fremont, CA, USA) with 10,000 events per sample. No autofluorescence was observed.

### RNA extraction

Mtb strains were grown in 10 mL square bottles in a shaking incubator at 37°C to reach OD_600nm_ ~ 0.8. Bacteria were harvested by centrifugation at 3,000 × *g* for 10 min at 4°C and washed once with cold sterile PBS. Total RNA was extracted using a FastRNA Pro Blue kit (MP) according to the manufacturer’s instructions. The samples were treated with RNase-free DNase (Promega) for 3 h at 37°C to remove contamination of genomic DNA. The samples were purified with RNeasy columns (Qiagen) according to the manufacturer’s instructions. The quantity and quality of RNA were assessed using a NanoDrop 1000 spectrophotometer (Thermo Fisher Scientific).

### qRT-PCR

cDNA was produced from each RNA sample using the reverse transcriptase kit (Qiagen) as per the manufacturer’s instructions. qRT-PCR was performed to quantify the expression levels of genes using the following primers: rv2702: forward primer, CCCTGGCTTAGTGGTACTGC; reverse primer, GTTGGGTATCAACGTCCCGT and rv0650: forward primer, CCTATCGACCTACACAGCGG; reverse primer, ACACCACCAAACCCAACAGA.

The expression levels of 16S rRNA were used as an internal control. Data were retrieved and processed with Microsoft Excel. The ΔΔ_CT_ values were calculated for the genes to be tested in each sample using untreated bacteria as the control, and the fold changes of the expression levels were calculated as 2^(−ΔΔ^_^CT^_^)^.

### Statistical analysis

The data are presented as the means ± SDs from two biological replicates and at least two technical replicates per condition. Unpaired two-tailed Student’s *t*-tests were used to compare the data, and *P* < 0.05 was considered significant.

### Biological safety considerations

The bacteria were handled within a Class-I safety-level cabinet in a containment level 3 laboratory.

## Data Availability

All data presented in this study are contained within the article and available upon request from the corresponding author. The metabolomics data generated in this study have been deposited in the MetaboLights repository (28) with the study identifier MTBLS13438.

## SUPPLEMENTAL MATERIAL

Supplemental material jb.00343-26-s0001.docx Fig. S1 to S9; Tables S1 to S3.

Supplemental text jb.00343-26-s0002.docx Supporting material.

Table S4 jb.00343-26-s0003.xlsx Metabolites abundances batch corrected.

ASM does not own the copyrights to Supplemental Material that may be linked to, or accessed through, an article. The authors have granted ASM a non-exclusive, world-wide license to publish the Supplemental Material files. Please contact the corresponding author directly for reuse.
