# Supplementary material for: Rv3400 is a phosphoglucomutase required for trehalose metabolism in Mycobacterium tuberculosis
Source: J Bacteriol. 2026 Sep 3;208(10):e00343-26. doi: 10.1128/jb.00343-26 (PMC13689574; doi:10.1128/jb.00343-26)
Supplement: Supplementary file 1 [file jb.00343-26-s0001.docx]

**Supporting Figures**

**S1:** 1D ^1^H NOESY spectral comparison of (a) Wildtype and (b) Knockout samples. Rectangular dotted boxes indicate additional resonances observed in the Knockout samplethat are absent in the Wildtype sample. The crossed blue line at 4.7 ppm marks removed water signal.

**S2:** 2D JRES spectra (blue) overlaid with 1D ^1^H NMR spectra (red ) from both Wildtype and Knockout samples. Red circles highlight the presence of additional spectral resonances present in the Knockout samples but absent in the Wildtype.

**S3:**  1D ^1^H NOESY NMR spectral overlay showing the spiking experiment results. The blue spectrum represents the Knockout sample without spiking, while the yellow, red, and green spectra correspond to the samples spiked with β-D-glucose-1-phosphate at concentrations of 30 µM, 60 µM, and 90 µM, respectively. Assignments for all the protons of β-D-glucose-1-phosphate are provided with spectral expanisons.

**S4:** 2D JRES spectra (blue) overlaid with 1D ^1^H NMR spectra (red) from the Knockout sample and the chemical standard β-D-Glucose-1-Phosphate. Red circles in the Knockout sample spectra highlight resonances that are identical resonances to those observed in the chemical standard β-D-glucose-1-phosphate spectra.

**S5:** Comparison of 2D COSY and 2D TOCSY NMR spectra between the Knockout sample and β-glucose-1-Phosphate. The figures highlight the presences of β-D-Glucose-1-Phosphate resonances in the Knockout sample, indicated by red circles

**S6. ROS response after 28 days starvation in PBS 0.5% Tyloxapol.** ROS were quantified by flow cytometry using CellROX deep red.

**S7:** Growth curves of wild type (black), Δrv3400 (red), complemented (grey) strains with no carbon source, 0.05%, 0.2% or 0.5% glucose or trehalose as the sole carbon source and using standard BSA.

**S8:** Growth curves of wild type (black), Δrv3400 (red), complemented (grey) strains with no carbon source, 0.05%, 0.2% or 0.5% glucose or trehalose as the sole carbon source and using BSA fraction V fatty acid free.

**S9:** **Deletion of Rv3400 leads to increase in susceptibility to H_2_O_2._** (**A**). Bar charts displaying number of viable bacteria expressed as CFU/mL for the wild type: back bars, Δ*rv3400*: red bars, completed: grey bars after 1 hour for untreated (UT), or H_2_O_2_ treated bacteria and for the wild type: dashed back bars, Δ*rv3400*: dashed red bars, completed: dashed grey bars when using glucose as sole carbon source. (**B**). Bar charts displaying number of viable bacteria expressed as CFU/mL for the wild type: back bars, Δ*rv3400*: red bars, completed: grey bars after 1 hour for untreated (UT), or H_2_O_2_ treated bacteria and for the wild type: dashed back bars, Δ*rv3400*: dashed red bars, completed: dashed grey bars when using glucose as sole carbon source. Asterisks indicate levels of significance. The significance levels of difference were determined by unpaired Student’s *t*-tests, with *: *p* < 0.05; **: *p* < 0.01.

**Table S1:** **qRT-PCR on the two glucokinases genes *rv0650* and *rv2702* compared to the wild type strain.**  The data are presented as the means±SDs from two biological replicates and three technical replicates. Using unpaired Student’s *t*-tests, the data are not significant compared to the wild type strain.

| Strain | *rv0650* | *rv2702* |
| --- | --- | --- |
| Δrv3400 | -0.29±0.55 | 0.91±0.96 |
| Δrv3400::rv3400 | 0.84±0.62 | 0.49±0.57 |
| Δrv3400::rv3400 D29A | 0.14±0.23 | 0.82±0.58 |

**Table S2:** **Percentage of ^13^C labelled metabolites.** The data are presented as the means±SDs from two biological replicates and three technical replicates.

| **Strains** | **glucose-6-phosphate** | **fructose-6-phosphate** | **β-glucose-1-phosphate** | ***p* value** |
| --- | --- | --- | --- | --- |
| wt | 79.1±2.4 | 80.8±2.1 | 61.6±2.3 |  |
| ΔRv3400 | 73.0±1.6 | 73.0±1.2 | 25.0±2.1 | 5.8E-11 |
| comp. | 70.7±1.3 | 72.3±0.9 | 58.6±1.5 |  |
| D29A | 71.6±1.8 | 71.4±0.6 | 25.2±1.2 | 1.8E-10 |

**Table S3: List of metabolites measured by LC-MS in the negative ion mode used this study.**

| **Name** | **Formula** | **Mass** | **[M-H]-** | **RT (mins)** |
| --- | --- | --- | --- | --- |
| Glycine | C2 H5 N O2 | 75.03203 | 74.02423 | 3.9 |
| L-Phenylalanine | C9 H11 N O2 | 165.07898 | 164.07118 | 6.1 |
| L-Valine | C5 H11 N O2 | 117.07898 | 116.07118 | 2.7 |
| L-Glutamine | C5 H10 N2 O3 | 146.06914 | 145.06134 | 4.7 |
| L-Glutamate | C5 H9 N O4 | 147.05316 | 146.04536 | 6.9 |
| L-Tyrosine | C9 H11 N O3 | 181.07389 | 180.06609 | 2.8 |
| L-Threonine | C4 H9 N O3 | 119.05824 | 118.05044 | 3.9 |
| L-Serine | C3 H7 N O3 | 105.04259 | 104.03479 | 4.9 |
| L-Proline | C5 H9 N O2 | 115.06333 | 114.05553 | 2.8 |
| L-Histidine | C6 H9 N3 O2 | 155.06948 | 154.06168 | 4.9 |
| L-Aspartic Acid | C4 H7 N O4 | 133.03751 | 132.02971 | 6.8 |
| L-Alanine | C3 H7 N O2 | 89.04768 | 88.03988 | 4.2 |
| mycothiol | C17 H30 N2 O12 S | 486.15195 | 485.14415 | 4.8 |
| mycothione | C34 H58 N4 O24 S2 | 970.28824 | 969.28044 | 12.2 |
| Ergothioneine | C9 H15 N3 O2 S | 229.0885 | 228.0807 | 1.8 |
| Glucose 6-phosphate | C6 H13 O9 P | 260.02972 | 259.02192 | 11.3 |
| Fructose 6-phosphate | C6 H13 O9 P | 260.02972 | 259.02192 | 9.6 |
| β-Glucose 1-phosphate | C6 H13 O9 P | 260.02972 | 259.02192 | 10.8 |
| Lactate | C3 H6 O3 | 90.03169 | 89.02389 | 2.3 |
| Pyruvate | C3 H4 O3 | 88.01604 | 87.00824 | 5.9 |
| Malate | C4 H6 O5 | 134.02152 | 133.01372 | 8.5 |
| Fumarate | C4 H4 O4 | 116.01096 | 115.00316 | 1.5 |
| Succinate | C4 H6 O4 | 118.02661 | 117.01881 | 7.6 |
| Oxoglutarate | C5 H6 O5 | 146.02152 | 145.01372 | 7.0 |
| trans-Aconitate | C7 H14 N2 O3 | 174.10044 | 173.09264 | 7.4 |
| Trehalose | C12 H22 O11 | 342.11621 | 341.10841 | 4.9 |
| Trehalose 6-phosphate | C12 H23 O14 P | 422.08254 | 421.07474 | 11.8 |
| Citrulline | C6 H13 N3 O3 | 175.09569 | 174.08789 | 5.7 |
| N2-Acetyl-L-ornithine | C7 H14 N2 O3 | 174.10044 | 173.09264 | 4.2 |
| Glycerylphosphorylethanolamine | C5 H14 N O6 P | 215.05587 | 214.04807 | 6.1 |
| 3',5'-cAMP | C10 H12 N5 O6 P | 329.05252 | 328.04472 | 2.5 |
| Inosine | C10 H12 N4 O5 | 268.08077 | 267.07297 | 4.9 |
| 5'-Adenosine monophosphate | C10 H14 N5 O7 P | 347.06308 | 346.05528 | 6.9 |
| UDP-GlcNAc | C17 H27 N3 O17 P2 | 607.08157 | 606.07377 | 7.1 |
| L-2-Aminoadipic acid | C6 H11 N O4 | 161.06881 | 160.06101 | 5.7 |
| Diaminopimelic acid | C7 H14 N2 O4 | 190.09536 | 189.08756 | 4.7 |

**Table S4: Metabolites abundances batch corrected**
